# Atypical Thrombotic Thrombocytopenic Purpura Presenting as Stroke

**DOI:** 10.1155/2019/7425320

**Published:** 2019-01-14

**Authors:** Pradeepthi Badugu, Modupe Idowu

**Affiliations:** Division of Hematology, Department of Internal Medicine, McGovern Medical School, The University of Texas Health Science Center at Houston, 6411 Fannin Street, Houston 77030, USA

## Abstract

Here we report a case of atypical thrombotic thrombocytopenic purpura that presented as an ischemic cerebrovascular accident. A 56-year-old man with multiple cardiovascular risk factors presented with sudden left-sided weakness, slurred speech, and left facial droop. He showed mild improvement when he was treated with thrombolytic therapy according to the hospital stroke protocol. Later in the course, he developed thrombocytopenia followed by schistocytes revealed by peripheral blood smear and other lab abnormalities. Thrombotic thrombocytopenic purpura (TTP) was suspected, and he was treated with total plasma exchange that improved his condition significantly. This case shows that TTP can have unusual and atypical presentations either with the first episode or upon relapse, making diagnosis extremely difficult. Because patients may not present the expected clinical findings, it is important to be aware of variant presentations. In the early stages of the disease, platelet aggregation and thrombus formation may not be widespread, and thrombocytopenia and microangiopathic hemolytic anemia may not be clinically evident. Patients can present soon after the onset of symptoms when the typical laboratory abnormalities may not have had ample time to manifest. Although most other similar cases in the literature had a previous typical presentation of the disease before an atypical presentation, our patient's first presentation was atypical. An atypical presentation of disease in a patient with cardiovascular risk factors may therefore be extremely difficult to diagnose. We believe that TTP should be considered for any patient presenting with stroke and thrombocytopenia.

## 1. Introduction

Thrombotic thrombocytopenic purpura (TTP) is often described as a pentad of thrombocytopenia, anemia, fever, renal dysfunction, and neurological abnormalities caused by unregulated von Willebrand factor- (VWF-) dependent platelet thrombosis [1]. ADAMTS13 is a metalloprotease that inhibits VWF-dependent platelet aggregation. Lack of this enzyme leads to persistent microvascular thrombi in multiple organs that in turn leads to the characteristic clinical presentations of TTP. Microthrombi exert a shearing force on the blood vessels and break red blood cells, resulting in schistocytes in peripheral smears. Clinical presentations of TTP can be typical or atypical either on initial presentation or upon relapse. Gastrointestinal, neurological, and bleeding manifestations are the most common presenting features of TTP [2]. “Atypical TTP” is the term used when clinical features at the time of admission are not typical of TTP but patients are later diagnosed with the disease. In atypical presentations, the manifestations of thrombosis may precede overt thrombotic microangiopathy and hemolytic anemia by days to months [3]. Here, we describe a case of atypical TTP that presented as an ischemic cerebrovascular accident without any hematological changes initially but later developed into thrombocytopenia and microangiopathic hemolytic anemia (MAHA). The patient was treated with fibrinolytics at presentation as part of a standard stroke protocol. However, his neurological symptoms improved only upon total plasma exchange.

## 2. Case

A 56-year-old man with a history of dyslipidemia, multiple transient ischemic attacks (TIAs), and a 40 pack-year smoking history presented to the local hospital with sudden left-sided weakness, slurred speech, and left facial droop. His family history was significant for stroke and diabetes in multiple family members. He was on atorvastatin for dyslipidemia. He had no history of connective tissue or autoimmune disease. He was diagnosed with acute ischemic cerebrovascular accident and was given tissue plasminogen activator (tPA). The patient received a single dose of atorvastatin 80 mg and aspirin 325 mg orally during the admission; he never received clopidogrel or ticlopidine therapy. The symptoms improved gradually, but he developed thrombocytopenia that worsened over the next few days. The team discontinued statin and aspirin therapy once they observed low platelets. The patient was managed conservatively, but his platelet counts reached a nadir at 16,000 per cubic mm. His peripheral blood smear showed no schistocytes in high-power fields. He was given a platelet transfusion with no improvement. He was then transferred to our hospital, and the inpatient hematology team was consulted. At the time of presentation, the patient complained of clumsiness in his left arm, although he was able to carry out daily activities with minimal difficulty. He was stable, the facial droop had resolved and speech slightly improved with residual dysarthria and expressive aphasia, and his motor power was better. A review of outside laboratory results revealed that 2 days before his transfer, his platelet count was 115,000 per cubic mm, BUN was 16, and his creatinine was 1.3. His initial lab values on arrival at our hospital were as follows: platelets 26,000 cells per cubic mm, hemoglobin (Hb) 10.7 g/dl, hematocrit (Hct) 31.2/L, leucocytes 16,300 cells per cubic mm, blood urea nitrogen 35, blood urea nitrogen/creatinine 27, lactic acid dehydrogenase of 794 (98–192), bilirubin 0.9, and reticulocyte count 2.8. During admission, B12 and folic acid levels, antiphospholipid panel, disseminated intravascular coagulation panel, coagulation panel, antinuclear antibodies, and rheumatoid factor were measured and found to be within normal limits. HIV, hepatitis B, and direct Coombs tests were negative. However, his peripheral blood smear showed 5-6 schistocytes/high-power field. Neither the patient nor his family members underwent any screening for connective tissue or autoimmune disorders.

The patient was immediately started on a daily total plasma exchange (TPE). On day 3, his neurological symptoms improved significantly, and his platelet count normalized to 167,000 per cubic mm. Despite the improved platelet count, TPE was continued for 2 more days. By day 5, his speech was clear and his expressive aphasia resolved. He recovered full strength in his right extremities and TPE was stopped. Blood samples were sent to the Blood Center of Wisconsin, Milwaukee, for measurement of ADAMTS13 levels, and we got the results 5 days later. ADAMTS13 activity measured using FRETS-VWF73 substrate was <5% (reference range ≥67%). ADAMTS13 inhibitor was measured using mixing studies with standard pooled plasma, and residual ADAMTS13 activity measured using FRETS-VWF73 substrate was <0.4 inhibitor units (reference range ≤0.4). Platelet count remained stable throughout the patient's stay. At the time of discharge, his counts and Hb/Hct remained stable, lactate dehydrogenase (LDH) was 249, and no schistocytes were present in peripheral blood smears. Because of his atypical presentation of TTP, he was advised to seek medical attention immediately if his neurological symptoms worsen to evaluate TTP relapse. After discharge, he was evaluated twice weekly with a CBC and LDH measurements after discharge for 2 weeks and once a week afterward. Higher levels of LDH were noticed subsequently normalized in 2 months. He complained of intermittent left facial numbness and tingling of the left hand during follow-up visits. The patient remained in remission without a relapse for 2 years after his initial presentation.

## 3. Literature Review

There are a few similar cases in the literature that are atypical presentations of TTP that presented as cerebrovascular accident (CVA). One is a 40-year-old woman with chronic relapsing TTP presented with facial numbness, left upper quadrant weakness, dysarthria, and diplopia. On admission, her Hb was 9.3 g/dl, PLT-239/cubic mm, and LDH-296 U/l (*N* = 68–239 u/l) and rare schistocytes were observed on a peripheral blood smear. A brain MRI showed acute right hemisphere infarction. Because of her significant history, TTP was considered as the etiology of her stroke, and she was started on TPE. After 12 hours of TPE, her hand and leg strength increased, and her dysarthria improved. Her neurological status significantly improved after 7 TPE treatments. Her ADAMTS13 level was 13% at the time of presentation, and ADAMTS13 inhibitor was detected [4]. Another case described by the same author is of a 42-year-old woman with a chronic history of relapsing TTP and a history of strokes in the setting of TTP. Her fourth episode of stroke was associated with motor weakness, dysphagia, and confusion, without lab findings for TTP. A magnetic resonance angiogram MRA showed an acute infarct in the left basal ganglia and old cerebral infarcts. Because of her history and stroke in the setting of TTP, she was immediately started on TPE. Her ADAMTS13 levels were 12% on admission, and she had ADAMTS13 IgG antibody. Her neurological symptoms significantly improved before the discontinuation of TPE, but she had multiple relapses in the following 3 years [4]. Tsai and Shulman [5] described a case of a 36-year-old African American woman with a first episode of TTP at the age of 27. She did well for 8 years, after which she experienced a stroke with motor deficits and dysarthria. Her platelet count, bilirubin, and LDH were normal, and no schistocytes were detected on the peripheral blood smear at the time of presentation. Three weeks later, she had an overt episode of TTP with PLT count 6 per cubic mm and schistocytes on the peripheral blood smear. Her neurological status and counts improved with TPE and prednisone. Idowu and Reddy [6] describe a case of a 45-year-old African American woman with a baseline of mild dysarthria, minimal expressive aphasia, and right upper extremity weakness and spasticity from a CVA and TTP 6 years previously. She now presented to the emergency department with left facial droop, left hemiparesis, and aphasia suggestive of stroke. She was diagnosed with acute stroke involving the right middle cerebral artery and started on tPA. Intra-arterial thrombolysis and thrombectomy were also performed but did not produce any improvement. Her hemoglobin count was normal with a slightly decreased platelet count, LDH was mildly elevated, and renal function was normal. A peripheral blood smear did not show any schistocytes. On the third day of hospitalization, her platelet count decreased from 113,000 to 97,000 cells per cubic mm, and hemoglobin dropped from 13.2 to 11.1 g/dl, without microangiopathic blood smear. TPE was started immediately. ADAMTS13 activity measured 16%, and mixing studies and ELISA assay were negative for ADAMTS13 inhibitor and IgG antibody. After 5 TPE treatments, she showed significant neurological recovery, stable Hb/Hct, and normal LDH, and her platelet count improved to 225,000 per cubic mm.

## 4. Discussion

The incidence of TTP is approximately 3.7 per million per year [7, 8]. Most cases are idiopathic, but some are associated with HIV, pregnancy, autoimmune diseases, infections, bone marrow transplantation, and use of drugs like cyclosporine, mitomycin, and ticlopidine [8]. In most cases, TTP is caused by deficiency of ADAMTS13. VWF is naturally secreted in ultralarge multimeres from endothelial cells. ADAMTS13 is a disintegrin and metalloprotease with thrombospondin type 1 motifs occurring in plasma that cleaves VWF at the Tyr 1605-Met 1606 bond in the central A2 subunit and prevents the accumulation of these large multimers that cause platelet aggregation and microvascular thrombosis [8]. A complete protease deficiency due to missense or frameshift mutations in the ADAMTS13 gene in the absence of an inhibitor is seen in familial TTP (Upshaw–Schulman syndrome). Patients with nonfamilial TTP have an acquired deficiency due to autoantibodies inhibiting the activity of the enzyme that accounts for most of the cases [7, 9, 10]. Deficiency of ADAMTS13 alone may not be the cause of TTP because it is observed that even with very low levels of the enzyme, the disease never manifested; hence, environmental factors play an important role in the development of the disease [3, 7, 11]. In the presence of ADAMTS13 deficiency, the tendency for VWF and platelets to form microthrombi is dependent on the platelet count and the conformational response of VWF to shear stress. Factors that increase shear stress activate VWF by causing conformational changes in the molecule. Environmental factors such as infections, inflammation, surgery, and pregnancy may decrease plasma ADAMTS13 activity by affecting its synthesis or by increasing its inactivation [11].

Clinical presentations of TTP are due to the microthromboses, constituting platelets, and VWF in the small vessels. Microthrombi cause shearing of the red blood cells resulting in schistocytes in peripheral smear, increased LDH, and decreased haptoglobin [3]. Although the disease is described as a pentad, the initial symptoms of TTP are nonspecific and variable [12, 13]. It is therefore risky to wait for the entire pentad to develop. Moreover, the classic pentad is not relevant to current practice. Often patients present with a range of neurological symptoms, including confusion, headaches, seizures, blurred vision, focal abnormalities, aphasia, stroke, and coma, due to the diffuse involvement of microcirculation in the brain [2, 3]. Some patients develop gastrointestinal symptoms such as abdominal pain, nausea, vomiting, and diarrhea that may be related to intestinal ischemia [2]. Renal abnormalities such as oliguria, microscopic hematuria, albuminuria, and acute renal failure may be involved [7]. Other possible symptoms are skin and mucosal bleeding, fatigue, dyspnea, and chest pain [2]. Thrombocytopenia with MAHA without an apparent cause is now sufficient to establish diagnosis and initiate treatment [2, 12]. In the early stages of the disease, platelet aggregation and thrombus formation may not be widespread, and thrombocytopenia and MAHA may not be clinically evident [4]. Patients can present soon after the onset of symptoms before classic laboratory abnormalities such as thrombocytopenia, and MAHA may not have sufficient time to manifest.

Neurological manifestations of TTP can be mistaken for acute ischemic stroke, as in the case presented here. Our patient had strong cardiovascular risk factors, tobacco use, dyslipidemia, and history of TIA; hence, CVA was rightly suspected. Moreover, his initial laboratory values showed very mild thrombocytopenia but no schistocytes on the peripheral blood smear, further hindering the early recognition of the disease. Thrombolysis, the treatment of CVA, might have led to severe hemorrhagic complications in the patient; fortunately, he did not have such complications. On the contrary, successful use of thrombolytic therapy in TTP patients with stroke has been described in the literature [6].The patient was transferred to our center because of worsening thrombocytopenia, and we suspected that he might have developed atypical thrombocytopenia even before his arrival to our facility and the examination of his peripheral blood smear. Our higher index for suspicion for atypical TTP was based on our experience with similar cases.

ADAMTS13 activity and autoimmune inhibitors against ADAMTS13 can be measured by lab assays. There are direct and indirect assays for the detection of ADAMTS13 activity, among which the direct FRETS-VWF73 is relatively rapid and simple. ELISA is considered to be more sensitive than an inhibitor assay for detecting ADAMTS13 autoantibodies [14]. Severe acquired ADAMTS13 deficiency is defined as a level less than 10%. Although it is specific for TTP, it is not necessary for diagnosis [2]. The severity of its deficiency in patients with idiopathic TTP corresponds neither to the clinical presentation nor to the response to treatment with plasma exchange. The poor correlation between the severity of disease and the levels of ADAMTS13 still needs to be explained [2, 6, 12, 14]. It may be related to the sensitivity of current assays [2, 7]. A prompt recognition and diagnosis of TTP among the patients presenting with stroke is important, as therapeutic plasma exchange provides immediate improvement but delay in diagnosis, and treatment can be catastrophic.

Although most of the other cases we described from the literature had previous episodes of TTP before an atypical presentation, our patient had an atypical presentation without any earlier history of TTP. Hence, we should have a high index of suspicion for TTP in any patient presenting with stroke and thrombocytopenia. Moreover, empirical plasma exchange should be considered if the patient does not have risk factors for stroke or if thrombocytopenia is persistent or worse without a clear reason. Plasma exchange is the recommended treatment for TTP [2, 12]. The survival rate was 10% before the advent of plasma exchange but is 78% with plasma exchange and 51% with plasma infusion [2]. Corticosteroids and other immunomodulatory drugs should be considered in most patients with acquired TTP to suppress the autoantibodies against ADAMTS13. All patients should be evaluated for possible contributing factors such as underlying infection, autoimmune disease, use of complicating drugs, and familial predisposition. Treatment of underlying condition should be promptly initiated, and any suspected offending drugs should be discontinued. All patients should be on folic acid supplementation. Assessment of baseline B12 levels and iron studies should be considered to ensure that the patient's reserve is adequate to mount a hematopoietic response to anemia. Response to treatment should be assessed by platelet count, and plasma exchange can be performed when platelet count normalizes and remains stable for 2 days, after which the patient can be discharged if clinically stable. After discharge, patients should be evaluated twice weekly for 2 weeks and once a week for another 2 weeks. Estimated risk of relapse for TTP is 41% for 7.5 years and is more common in the first year after occurrence of TTP [2]. The risk of relapse increases when the levels of ADAMTS13 are persistently less than 10% or when the levels continue to decrease during remission. In such a scenario, treatment with rituximab should be considered [11]. Studies indicate that patients with high titers of inhibitors of ADAMTS13 have a delayed response to therapy, high risk of complications, and higher rates of relapse [7]. Many patients have persistent minor cognitive abnormalities even after recovery [14].

In conclusion, TTP should be considered in all patients who develop CVA or TIA and unexplained persistent thrombocytopenia even when they have cardiovascular risk factors. It is imperative to have a high index of suspicion for TTP in this context because delay in diagnosis may lead to death and because early recognition of TTP and proper treatment can lead to rapid improvement of symptoms and can prevent unnecessary therapy resulting in further complications. Empiric therapy with plasma exchange may be appropriate for patients with suspected atypical presentation while awaiting the results of ADAMTS13 tests. Thrombolytic therapy may lead to life-threatening bleeding complications in these patients. TTP can have unusual and atypical presentations that may make the diagnosis extremely difficult, as the patients may lack the expected clinical findings; thus, it is important to be aware of its range of presentations. Atypical presentation of TTP in a patient with cardiovascular risk factors may complicate diagnosis, but a high index of suspicion may save lives.
